# Supplementary material for: A Likelihood Approach for Real-Time Calibration of Stochastic Compartmental Epidemic Models
Source: PLoS Comput Biol. 2017 Jan 17;13(1):e1005257. doi: 10.1371/journal.pcbi.1005257 (PMC5240920; doi:10.1371/journal.pcbi.1005257)
Supplement: S1 File — (TAR.GZ) [file pcbi.1005257.s014.tar.gz › HSPH_Online-SI-Revision/MSS10/n1-extreme/n1-extreme_NEW2_table8.pdf]

1	newly 3 weeks spec	5.	0.43	{0., 1.}	91.4	1.34836	newly 3 weeks cumul	23.	2.19	{1., 4.}	90.4783	1.12836	newly 5 weeks spec	0.1	0.23	{0., 1.}	296.	0.186812	newly 5 weeks cumul	25.	2.75	{1., 5.}	89.	1.01924	newly 52 weeks cumul	29.	3.12	{1., 5.}	89.2414	1.04503
2	newly 3 weeks spec	4.	5.19	{2., 9.}	53.25	0.213999	newly 3 weeks cumul	26.	20.24	{17., 24.}	22.5385	0.114943	newly 5 weeks spec	4.	2.66	{1., 5.}	43.	0.313831	newly 5 weeks cumul	34.	26.75	{22., 32.}	21.3235	0.107661	newly 48 weeks cumul	44.	36.73	{32., 41.}	17.25	0.0840618
3	newly 3 weeks spec	13.	10.88	{6., 16.}	26.6154	0.149741	newly 3 weeks cumul	58.	45.83	{28., 59.}	22.1552	0.123227	newly 5 weeks spec	7.	5.61	{3., 10.}	37.5714	0.216422	newly 5 weeks cumul	73.	58.85	{40., 74.}	20.2329	0.110042	newly 50 weeks cumul	89.	74.89	{53., 90.}	16.6854	0.0862877
4	newly 3 weeks spec	10.	17.09	{11., 23.}	72.3	0.221393	newly 3 weeks cumul	62.	72.93	{64., 84.}	19.0806	0.0746658	newly 5 weeks spec	6.	8.42	{4., 12.}	54.3333	0.185572	newly 5 weeks cumul	83.	93.76	{81., 108.}	15.3012	0.0611022	newly 49 weeks cumul	123.	114.3	{94., 133.}	11.7236	0.054918
5	newly 3 weeks spec	9.	10.44	{7., 14.}	31.3333	0.124372	newly 3 weeks cumul	33.	41.82	{38., 46.}	26.7879	0.101324	newly 5 weeks spec	4.	5.09	{2., 9.}	52.25	0.208874	newly 5 weeks cumul	44.	54.2	{49., 59.}	23.1818	0.0893886	newly 42 weeks cumul	59.	68.	{63., 74.}	15.2881	0.0609804
6	newly 3 weeks spec	11.	13.42	{8., 18.}	32.9091	0.124001	newly 3 weeks cumul	43.	59.64	{51., 67.}	38.6977	0.139186	newly 5 weeks spec	6.	5.61	{2., 10.}	38.5	0.2022	newly 5 weeks cumul	53.	74.49	{63., 84.}	40.5472	0.144997	newly 47 weeks cumul	71.	86.68	{73., 101.}	23.1831	0.087688
7	newly 3 weeks spec	6.	7.58	{3., 13.}	57.6667	0.239638	newly 3 weeks cumul	35.	32.39	{16., 49.}	35.4	0.17912	newly 5 weeks spec	5.	3.3	{1., 7.}	51.2	0.410361	newly 5 weeks cumul	52.	41.26	{21., 62.}	32.4231	0.189528	newly 51 weeks cumul	64.	50.23	{25., 78.}	33.0781	0.19535
8	newly 3 weeks spec	9.	0.5	{0., 1.}	94.4444	1.53414	newly 3 weeks cumul	27.	1.92	{1., 3.}	92.8889	1.22831	newly 5 weeks spec	1.	0.24	{0., 1.}	90.	0.849823	newly 5 weeks cumul	32.	2.53	{1., 5.}	92.0938	1.17655	newly 45 weeks cumul	35.	3.	{1., 6.}	91.4286	1.1613
9	newly 3 weeks spec	3.	6.69	{3., 10.}	127.	0.340167	newly 3 weeks cumul	16.	28.68	{21., 35.}	79.25	0.24642	newly 5 weeks spec	3.	3.2	{1., 6.}	52.	0.275579	newly 5 weeks cumul	22.	36.12	{28., 45.}	64.1818	0.208336	newly 52 weeks cumul	25.	41.83	{31., 55.}	67.4	0.21266
10	newly 3 weeks spec	17.	12.18	{8., 16.}	30.3529	0.171278	newly 3 weeks cumul	46.	50.18	{44., 56.}	11.5652	0.0468756	newly 5 weeks spec	5.	7.17	{4., 10.}	54.6	0.185266	newly 5 weeks cumul	57.	66.88	{60., 73.}	17.5439	0.0690732	newly 45 weeks cumul	77.	87.25	{78., 96.}	13.7013	0.0548494
11	newly 3 weeks spec	5.	4.83	{2., 8.}	38.6	0.214729	newly 3 weeks cumul	17.	22.88	{17., 29.}	37.4118	0.132058	newly 5 weeks spec	0.1	2.47	{0., 5.}	2390.	1.21605	newly 5 weeks cumul	19.	28.75	{20., 38.}	53.2105	0.176407	newly 51 weeks cumul	19.	33.19	{22., 45.}	76.5789	0.233802
12	newly 3 weeks spec	1.	2.01	{0., 5.}	155.	0.51723	newly 3 weeks cumul	17.	9.99	{5., 15.}	43.7059	0.284148	newly 5 weeks spec	2.	0.68	{0., 2.}	83.	0.947356	newly 5 weeks cumul	22.	11.77	{5., 19.}	48.3182	0.329808	newly 52 weeks cumul	22.	12.66	{5., 22.}	46.6364	0.317079
13	newly 3 weeks spec	20.	15.24	{10., 20.}	26.1	0.144094	newly 3 weeks cumul	80.	58.6	{49., 67.}	26.75	0.138049	newly 5 weeks spec	17.	11.03	{6., 16.}	37.2353	0.22463	newly 5 weeks cumul	116.	82.37	{71., 93.}	28.9914	0.151167	newly 45 weeks cumul	196.	124.62	{101., 150.}	36.4184	0.201261
14	newly 3 weeks spec	2.	2.45	{0., 5.}	82.5	0.42328	newly 3 weeks cumul	9.	12.03	{6., 27.}	54.5556	0.171199	newly 5 weeks spec	0.1	0.95	{0., 4.}	966.	0.530718	newly 5 weeks cumul	9.	14.71	{7., 35.}	74.7778	0.196645	newly 49 weeks cumul	9.	16.85	{7., 40.}	96.3333	0.230511
15	newly 3 weeks spec	2.	18.76	{14., 24.}	838.	0.963843	newly 3 weeks cumul	21.	71.1	{62., 78.}	238.571	0.527828	newly 5 weeks spec	1.	12.27	{7., 16.}	1127.	1.06774	newly 5 weeks cumul	25.	99.2	{90., 107.}	296.8	0.597581	newly 44 weeks cumul	29.	144.94	{128., 163.}	399.793	0.697011
16	newly 3 weeks spec	13.	4.28	{1., 7.}	67.0769	0.594562	newly 3 weeks cumul	45.	19.9	{12., 28.}	55.7778	0.376603	newly 5 weeks spec	6.	2.03	{0., 5.}	71.8333	0.846787	newly 5 weeks cumul	59.	24.92	{13., 36.}	57.7627	0.403274	newly 51 weeks cumul	82.	28.65	{14., 42.}	65.061	0.493836
17	newly 3 weeks spec	14.	19.77	{14., 26.}	43.7857	0.150788	newly 3 weeks cumul	63.	77.54	{68., 86.}	23.0794	0.0885675	newly 5 weeks spec	8.	12.54	{8., 18.}	62.25	0.20538	newly 5 weeks cumul	82.	106.03	{95., 118.}	29.3049	0.110196	newly 48 weeks cumul	132.	146.9	{133., 162.}	11.8939	0.0478156
18	newly 3 weeks spec	15.	11.14	{6., 15.}	29.3333	0.166103	newly 3 weeks cumul	37.	45.63	{40., 50.}	23.5946	0.0902233	newly 5 weeks spec	5.	5.95	{3., 9.}	37.8	0.154822	newly 5 weeks cumul	50.	59.92	{54., 66.}	20.08	0.0784598	newly 51 weeks cumul	60.	75.98	{70., 82.}	26.6333	0.101693
19	newly 3 weeks spec	10.	3.2	{0., 7.}	74.	0.827064	newly 3 weeks cumul	28.	13.85	{7., 19.}	67.0357	0.454857	newly 5 weeks spec	4.	1.37	{0., 3.}	72.75	0.863356	newly 5 weeks cumul	43.	17.53	{8., 23.}	69.4186	0.520599	newly 52 weeks cumul	65.	21.15	{9., 29.}	73.6769	0.626385
20	newly 3 weeks spec	4.	0.83	{0., 2.}	79.25	0.921342	newly 3 weeks cumul	15.	3.74	{2., 5.}	75.0667	0.645995	newly 5 weeks spec	2.	0.4	{0., 1.}	82.	0.97348	newly 5 weeks cumul	21.	4.65	{3., 6.}	77.8571	0.677435	newly 45 weeks cumul	30.	5.52	{3., 8.}	81.6	0.755547
21	newly 3 weeks spec	20.	21.64	{16., 27.}	18.9	0.0777501	newly 3 weeks cumul	78.	79.23	{71., 87.}	6.32051	0.0271079	newly 5 weeks spec	5.	15.5	{10., 21.}	210.	0.473791	newly 5 weeks cumul	98.	113.39	{101., 126.}	15.8878	0.0627126	newly 48 weeks cumul	119.	181.26	{155., 202.}	52.3193	0.180542
22	newly 3 weeks spec	4.	2.49	{0., 4.}	44.75	0.386958	newly 3 weeks cumul	28.	10.82	{7., 14.}	61.3571	0.428411	newly 5 weeks spec	5.	1.2	{0., 3.}	76.4	0.932075	newly 5 weeks cumul	41.	13.98	{10., 19.}	65.9024	0.481275	newly 49 weeks cumul	59.	17.42	{10., 24.}	70.4746	0.553274
23	newly 3 weeks spec	8.	14.64	{10., 20.}	84.25	0.251009	newly 3 weeks cumul	27.	64.77	{56., 74.}	139.889	0.377415	newly 5 weeks spec	5.	8.14	{3., 13.}	75.2	0.238637	newly 5 weeks cumul	38.	83.54	{72., 94.}	119.842	0.339707	newly 51 weeks cumul	59.	102.05	{83., 121.}	72.9661	0.233747
24	newly 3 weeks spec	5.	3.99	{1., 6.}	37.4	0.237132	newly 3 weeks cumul	19.	19.82	{16., 24.}	14.2105	0.0603568	newly 5 weeks spec	2.	1.62	{0., 3.}	57.	0.453741	newly 5 weeks cumul	25.	24.57	{19., 30.}	12.12	0.0541051	newly 52 weeks cumul	25.	27.56	{21., 34.}	16.56	0.0659578
25	newly 3 weeks spec	8.	6.83	{3., 11.}	34.875	0.188784	newly 3 weeks cumul	31.	31.58	{22., 40.}	17.871	0.0779271	newly 5 weeks spec	8.	3.34	{1., 6.}	61.	0.559077	newly 5 weeks cumul	48.	39.82	{29., 51.}	22.0417	0.113072	newly 49 weeks cumul	114.	48.79	{32., 67.}	57.2018	0.385718
26	newly 3 weeks spec	4.	1.46	{0., 3.}	68.	0.675239	newly 3 weeks cumul	13.	8.09	{5., 10.}	49.	0.282545	newly 5 weeks spec	1.	0.72	{0., 2.}	88.	0.652198	newly 5 weeks cumul	14.	9.78	{6., 12.}	45.5714	0.242546	newly 51 weeks cumul	19.	11.07	{7., 13.}	54.7895	0.32425
27	newly 3 weeks spec	15.	0.62	{0., 2.}	95.8667	1.89544	newly 3 weeks cumul	54.	13.64	{3., 28.}	74.7407	0.726406	newly 5 weeks spec	8.	0.02	{0., 0.}	99.75	1.88309	newly 5 weeks cumul	70.	13.77	{3., 28.}	80.3286	0.836636	newly 46 weeks cumul	110.	13.77	{3., 28.}	87.4818	1.03293
28	newly 3 weeks spec	9.	11.17	{7., 16.}	33.2222	0.124207	newly 3 weeks cumul	43.	45.42	{40., 51.}	8.7907	0.0363791	newly 5 weeks spec	3.	6.39	{3., 9.}	114.333	0.30395	newly 5 weeks cumul	53.	59.88	{54., 65.}	13.3585	0.0534547	newly 50 weeks cumul	70.	77.73	{72., 84.}	11.1286	0.0451612
29	newly 3 weeks spec	2.	0.9	{0., 2.}	61.	0.617076	newly 3 weeks cumul	14.	3.45	{2., 5.}	75.3571	0.654203	newly 5 weeks spec	0.1	0.55	{0., 1.}	560.	0.477093	newly 5 weeks cumul	14.	4.82	{3., 7.}	65.5714	0.483653	newly 47 weeks cumul	17.	6.16	{4., 8.}	63.7647	0.454913
30	newly 3 weeks spec	2.	7.72	{4., 11.}	287.	0.550164	newly 3 weeks cumul	16.	35.56	{29., 44.}	122.25	0.341344	newly 5 weeks spec	3.	3.	{1., 6.}	53.3333	0.323257	newly 5 weeks cumul	21.	43.65	{35., 53.}	107.857	0.311716	newly 42 weeks cumul	31.	49.91	{39., 61.}	61.129	0.199883
31	newly 3 weeks spec	4.	5.16	{1., 9.}	65.	0.282424	newly 3 weeks cumul	13.	23.49	{13., 36.}	83.3077	0.231289	newly 5 weeks spec	3.	2.43	{0., 5.}	56.3333	0.430865	newly 5 weeks cumul	20.	29.99	{17., 45.}	59.55	0.18584	newly 51 weeks cumul	27.	36.64	{19., 56.}	52.8889	0.180583
32	newly 3 weeks spec	1.	2.58	{1., 5.}	170.	0.39626	newly 3 weeks cumul	8.	12.88	{9., 16.}	62.	0.201181	newly 5 weeks spec	0.1	1.39	{0., 3.}	1350.	0.860751	newly 5 weeks cumul	8.	16.11	{12., 20.}	101.375	0.29426	newly 51 weeks cumul	10.	18.48	{13., 24.}	84.8	0.253497
33	newly 3 weeks spec	21.	17.54	{12., 22.}	20.2857	0.104527	newly 3 weeks cumul	67.	66.9	{60., 75.}	7.22388	0.0314051	newly 5 weeks spec	11.	11.04	{6., 16.}	26.1818	0.117324	newly 5 weeks cumul	89.	91.83	{83., 99.}	5.96629	0.0252409	newly 47 weeks cumul	124.	128.5	{116., 139.}	6.64516	0.0279315
34	newly 3 weeks spec	9.	12.27	{7., 17.}	46.5556	0.165231	newly 3 weeks cumul	31.	50.56	{43., 58.}	63.0968	0.210005	newly 5 weeks spec	2.	6.61	{3., 10.}	233.5	0.499903	newly 5 weeks cumul	36.	66.03	{59., 73.}	83.4167	0.261961	newly 50 weeks cumul	40.	83.42	{74., 93.}	108.55	0.317642
35	newly 3 weeks spec	11.	4.02	{1., 8.}	63.4545	0.554026	newly 3 weeks cumul	46.	15.6	{10., 20.}	66.087	0.48733	newly 5 weeks spec	5.	2.13	{0., 4.}	60.6	0.544098	newly 5 weeks cumul	59.	20.45	{15., 26.}	65.339	0.472991	newly 51 weeks cumul	68.	25.96	{16., 35.}	61.8235	0.433101
36	newly 3 weeks spec	10.	6.99	{4., 10.}	35.1	0.209594	newly 3 weeks cumul	26.	28.42	{24., 34.}	13.6154	0.05475	newly 5 weeks spec	7.	4.11	{1., 7.}	46.7143	0.356683	newly 5 weeks cumul	37.	38.15	{32., 44.}	10.7297	0.0453839	newly 48 weeks cumul	53.	48.76	{40., 59.}	13.434	0.0628035
37	newly 3 weeks spec	11.	3.46	{1., 6.}	68.5455	0.623396	newly 3 weeks cumul	35.	15.82	{12., 20.}	54.8	0.354161	newly 5 weeks spec	7.	1.73	{0., 4.}	75.5714	0.81081	newly 5 weeks cumul	50.	20.03	{15., 25.}	59.94	0.404447	newly 43 weeks cumul	55.	24.77	{18., 31.}	54.9636	0.355116
38	newly 3 weeks spec	6.	9.93	{6., 13.}	72.1667	0.24147	newly 3 weeks cumul	20.	41.93	{36., 48.}	109.65	0.319163	newly 5 weeks spec	2.	5.53	{2., 9.}	178.5	0.411391	newly 5 weeks cumul	28.	54.31	{48., 60.}	93.9643	0.285984	newly 48 weeks cumul	40.	67.32	{60., 74.}	68.3	0.224453
39	newly 3 weeks spec	6.	8.26	{4., 14.}	51.3333	0.170672	newly 3 weeks cumul	22.	37.29	{26., 55.}	69.5	0.20889	newly 5 weeks spec	2.	3.83	{1., 7.}	113.5	0.364015	newly 5 weeks cumul	28.	47.45	{33., 71.}	69.4643	0.208721	newly 51 weeks cumul	34.	58.45	{40., 90.}	72.0882	0.21017
40	newly 3 weeks spec	20.	16.63	{10., 21.}	19.65	0.106167	newly 3 weeks cumul	45.	66.91	{59., 75.}	48.6889	0.170414	newly 5 weeks spec	5.	9.84	{6., 14.}	98.8	0.282043	newly 5 weeks cumul	56.	88.58	{80., 95.}	58.1786	0.198012	newly 49 weeks cumul	62.	117.97	{106., 130.}	90.2742	0.27779
41	newly 3 weeks spec	17.	16.17	{11., 22.}	21.4706	0.0996482	newly 3 weeks cumul	35.	71.7	{63., 82.}	104.857	0.308903	newly 5 weeks spec	9.	8.07	{4., 12.}	32.5556	0.179226	newly 5 weeks cumul	50.	91.28	{79., 103.}	82.56	0.259147	newly 44 weeks cumul	61.	110.38	{89., 130.}	80.9508	0.253797
42	newly 3 weeks spec	1.	7.66	{3., 12.}	666.	0.826294	newly 3 weeks cumul	20.	31.95	{15., 43.}	70.45	0.22956	newly 5 weeks spec	1.	3.87	{1., 6.}	291.	0.539511	newly 5 weeks cumul	23.	41.07	{19., 52.}	85.3478	0.257305	newly 52 weeks cumul	25.	50.94	{24., 64.}	107.36	0.298427
43	newly 3 weeks spec	13.	9.17	{4., 15.}	38.5385	0.235797	newly 3 weeks cumul	49.	37.97	{27., 65.}	35.2449	0.181959	newly 5 weeks spec	1.	4.88	{2., 9.}	396.	0.660386	newly 5 weeks cumul	57.	49.51	{36., 83.}	31.1754	0.147269	newly 51 weeks cumul	68.	62.47	{44., 106.}	30.8088	0.138459
44	newly 3 weeks spec	6.	8.03	{5., 12.}	44.1667	0.157913	newly 3 weeks cumul	30.	31.45	{26., 36.}	11.1	0.0466561	newly 5 weeks spec	3.	4.84	{2., 8.}	76.6667	0.240414	newly 5 weeks cumul	42.	42.74	{37., 48.}	8.66667	0.0372182	newly 49 weeks cumul	55.	56.8	{47., 68.}	11.4545	0.0481535
45	newly 3 weeks spec	9.	7.98	{4., 13.}	28.8889	0.145359	newly 3 weeks cumul	41.	34.34	{28., 41.}	17.2683	0.0864319	newly 5 weeks spec	11.	4.07	{1., 7.}	63.	0.536966	newly 5 weeks cumul	64.	44.2	{37., 51.}	30.9375	0.164569	newly 52 weeks cumul	78.	54.7	{44., 65.}	29.8718	0.158901
46	newly 3 weeks spec	12.	6.2	{3., 9.}	50.5	0.346281	newly 3 weeks cumul	35.	24.23	{19., 29.}	34.8286	0.184971	newly 5 weeks spec	5.	3.59	{1., 7.}	44.2	0.300545	newly 5 weeks cumul	52.	32.5	{26., 37.}	40.6538	0.225895	newly 49 weeks cumul	69.	45.49	{35., 54.}	36.5652	0.199909
47	newly 3 weeks spec	16.	9.3	{6., 13.}	41.875	0.258443	newly 3 weeks cumul	65.	32.99	{28., 39.}	49.2462	0.298702	newly 5 weeks spec	11.	6.08	{3., 9.}	46.	0.319896	newly 5 weeks cumul	87.	46.67	{39., 55.}	46.3563	0.273825	newly 50 weeks cumul	118.	73.54	{58., 89.}	37.678	0.211438
48	newly 3 weeks spec	17.	9.87	{3., 16.}	43.8235	0.301915	newly 3 weeks cumul	49.	40.43	{26., 62.}	30.2653	0.160085	newly 5 weeks spec	7.	4.99	{2., 9.}	45.8571	0.347157	newly 5 weeks cumul	64.	52.69	{32., 79.}	31.2969	0.165707	newly 50 weeks cumul	85.	67.04	{37., 105.}	32.9882	0.182112
49	newly 3 weeks spec	8.	5.69	{3., 9.}	36.125	0.223758	newly 3 weeks cumul	31.	24.39	{18., 30.}	21.5806	0.112148	newly 5 weeks spec	3.	3.35	{1., 6.}	43.6667	0.211948	newly 5 weeks cumul	39.	31.96	{24., 38.}	18.6667	0.0943193	newly 52 weeks cumul	53.	40.34	{33., 48.}	24.	0.123482
50	newly 3 weeks spec	6.	5.54	{2., 10.}	42.	0.216126	newly 3 weeks cumul	38.	24.09	{17., 31.}	36.6053	0.209774	newly 5 weeks spec	6.	2.66	{0., 6.}	57.6667	0.589042	newly 5 weeks cumul	47.	30.93	{20., 41.}	34.3191	0.19675	newly 50 weeks cumul	63.	36.87	{22., 52.}	41.8571	0.255057
